# Beyond the Hype: A Scoping Review of TikTok's Potential and Pitfalls in Clinical Education

**DOI:** 10.1111/tct.70413

**Published:** 2026-04-11

**Authors:** Hester Lacey, Sara Donetto, Jim Price, Wajeeha Aziz

**Affiliations:** ^1^ Department of Medical Education Brighton and Sussex Medical School Brighton UK

## Abstract

**Introduction:**

Multimedia, including TikTok, is increasingly predominant for asynchronous and synchronous learning. This scoping review aimed to determine the role of TikTok for clinical education, including assessment of learning benefits, limitations, accessibility, acceptability and feasibility.

**Methods:**

We conducted a scoping review following Joanna Briggs Institute's (JBI) guidelines. Literature eligibility criteria included any literature discussing TikTok for clinical education. Findings were narratively synthesised on a bespoke data collection tool and analysed.

**Results:**

This review highlighted both strengths and risks relating to TikTok for clinical education. Identified strengths included benefits for student engagement and enjoyment from learning, improving accessibility and acceptability of learning content and opportunities for advocacy and outreach. The scope of educational opportunities included direct learning of clinical information, academic discourse and dissemination and professional networking. Learning benefits related to integrative multimedia pedagody and reduction of learner cognitive load, which optimised the learning potential from content viewed. Benefits detailed were predominantly conceptual, with a lack of studies analysing TikTok educational use and risk of bias from studies high. Risks and challenges relating to TikTok were notable, including cybersecurity, misinformation, addiction and exploitation risks from social media use, as well as a lack of professional guidance and legislation.

**Conclusions:**

While TikTok has potential, its integration into clinical education remains limited by risks. Ethical considerations limit the feasibility and justifiability of learner and educator use. Without formal legislation, governance, resource provision and training, the use of TikTok by clinical educators is practically unrealistic and offers unacceptable educational, academic, personal and professional risks to both learners and educators.

## 1 Introduction

Free Open Access Medical Education (FOAMed) describes the use of online and asynchronous clinical education resources, accelerated by the COVID‐19 pandemic [1]. Where previously basic tools like PowerPoint predominated, sociocultural technological dominance has driven the integration of audio‐visual content, simulation and virtual reality into clinical education [2]. Student technological literacy promotes accessibility and engagement, facilitating novel educational approaches for asynchronous, synchronous and self‐directed learning [3]. Audio‐visual tools offer dynamic alternatives to text‐heavy content, supporting diverse learning styles, with platforms like Zoom and MS Teams facilitating the incorporation of quizzes, polls and interactive discussions [3]. Well‐designed multimedia can enhance student engagement and accommodate different learning styles, with subsequent learning benefits [4]. Digital teaching also influences the hidden curriculum by shaping norms for online professionalism, mediating educator role modelling of digital codes of conduct in controlled, low‐risk settings [5].

### 1.1 TikTok for Clinical Education

Social media (SoMe), including YouTube, Instagram, Facebook and podcast streaming services, is increasingly popular for educators [6]. TikTok has emerged as a popular educational platform, offering short‐form, engaging content in the form of ‘micro‐learning’, where users can view 60‐s ‘shorts’ of high‐yield information, with the integration of audio, video, text and animations, covering topics from basic science, disease presentations and communication skills [7]. The hashtag #learnontiktok, launched in 2021, expanded educational content and was globally popularised during the COVID‐19 pandemic [8]. TikTok's 1.8 billion monthly active users and 246.1 million views under the hashtag #MedEd in early 2024 highlight its growing educational role [8]. Compared to X (formerly Twitter), which had 4.4 million posts in 2012–2022, TikTok dominates in engagement [9].

### 1.2 Multimedia Learning Pedagogy

**FIGURE 1:**
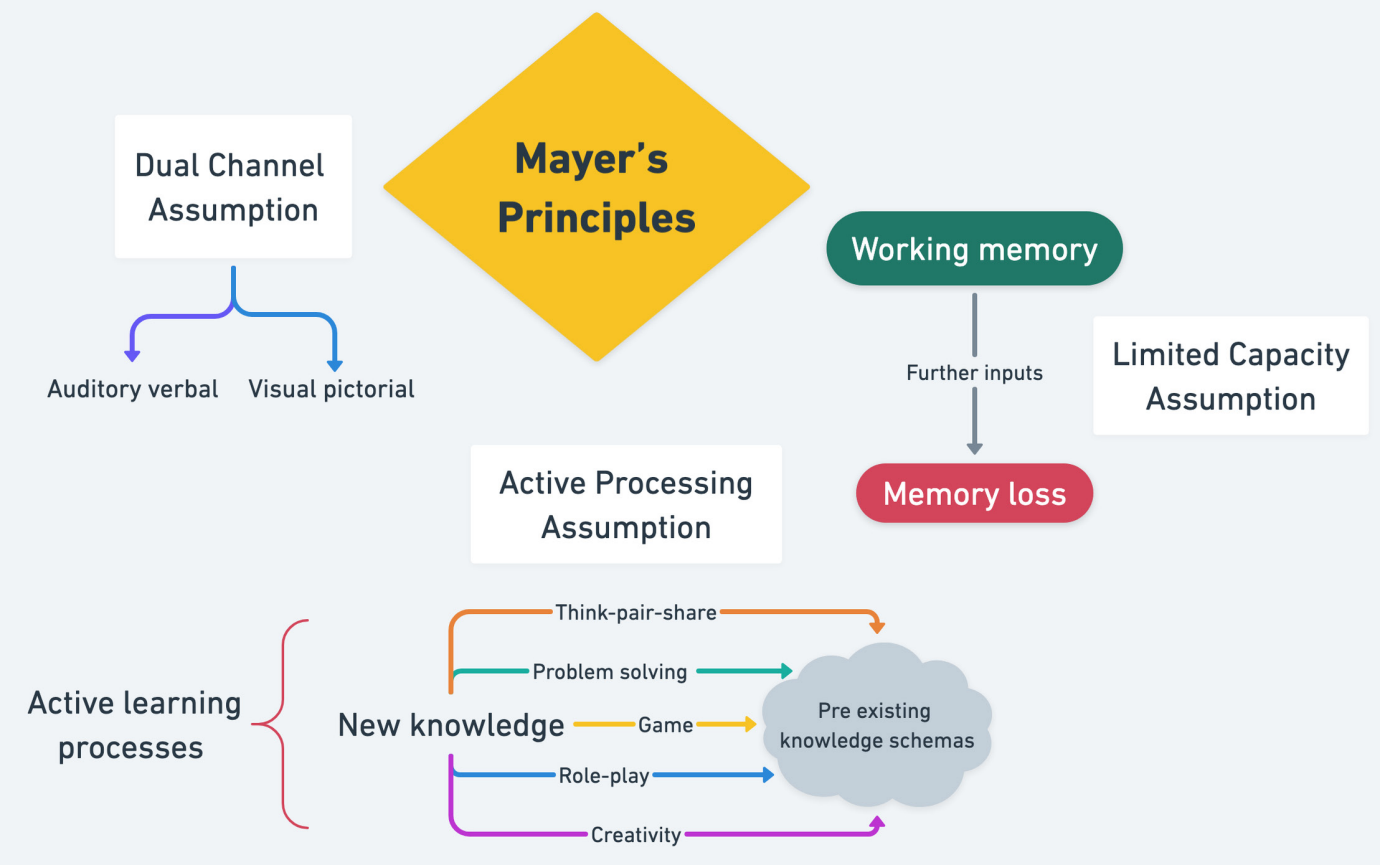
Mayer's principles of multimedia learning.

**FIGURE 2:**
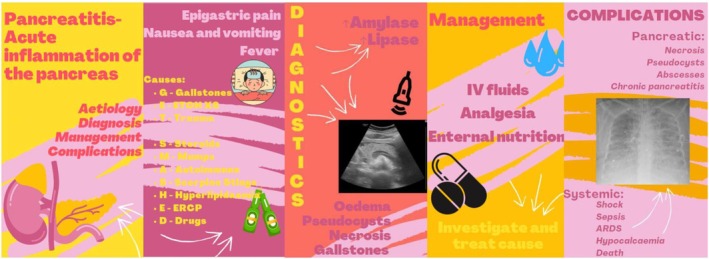
TikTok storyboard.

Different SoMe platforms have varied educational benefits. Engagement depends on student familiarity, platform accessibility and the ability to meet learning objectives from presented content [10]. Well‐designed multimedia improves engagement and comprehension, while poorly structured content can overload learners, reducing learning effectiveness [3]. Selecting the right platform is critical. YouTube is valuable for long‐form video content but lacks real‐time assessment features. TikTok, by contrast, can integrate video, audio and interactive tools for knowledge testing [2]. Educators must choose tools that maximise engagement and accessibility while limiting excessive platform switching that disrupts learning continuity [11]. Mayer's multimedia learning principles suggest that combining visual, auditory and interactive content enhances knowledge retention by facilitating information transfer from working to long‐term memory [3] (Figure 1). Cognitive load theory, integral to Mayer's principles, applies to TikTok's educational potential [2]. Effective content design minimises cognitive overload by excluding redundant information and emphasising key concepts [12]. TikTok's short format naturally reduces extraneous load, while strategic multimedia use enhances engagement and knowledge retention, which, if balanced with content complexity, can prompt effective learning (Figure 2) [13]. While TikTok's potential for clinical education is promising, empirical evidence remains limited [2]. Challenges include integration within existing curricula and resistance to new teaching methods [14, 15]. Moreover, content creators are increasingly capitalising on TikTok's popularity to monetise educational material; the rise of ‘Medical Influencers’, describing previously practising clinicians who have rebranded into SoMe personalities, brings both opportunities and risks, as content volume increases but quality varies, influenced by commercial biases and financial incentives [16]. Establishing clear academic benefits of TikTok and addressing these risks will determine its long‐term viability in medical education and help educators integrate it effectively in classroom settings, while mitigating the risks of credibility, safeguarding and misinformation [4].

### 1.3 Rationale for Scoping Review

TikTok's role in clinical education is primarily explored through narrative studies or small, heterogeneous experimental studies, limiting reliable conclusions [16]. While scoping reviews considering the role of multiple SoMe platforms, including TikTok, in clinical education exist, there are no comprehensive scoping or systematic reviews focusing solely on TikTok's benefits and challenges [17, 18]. A scoping review was performed to synthesize key themes, guide educators on its use and identify future research directions.

### 1.4 Objectives

This review aimed to establish the role, benefits and limitations of TikTok in clinical education, summarise existing literature and draw conclusions, identify knowledge gaps and determine areas for future research. Relevant literature includes any work discussing the role of TikTok for clinical education (both undergraduate, postgraduate and continuing professional education). Clinical education was considered to include all healthcare settings and all relevant fields of healthcare education.

## 2 Methods

This scoping review was conducted in line with the Joanna Briggs Institute's (JBI) guidelines for scoping reviews [19].

### 2.1 Protocol and Registration

An a priori protocol was developed to predefine the objectives, methods and outcomes. The PRISMA‐ScR checklist was used to structure reporting, according to JBI guidance (Appendix S1).

### 2.2 Eligibility Criteria

**FIGURE 3:**
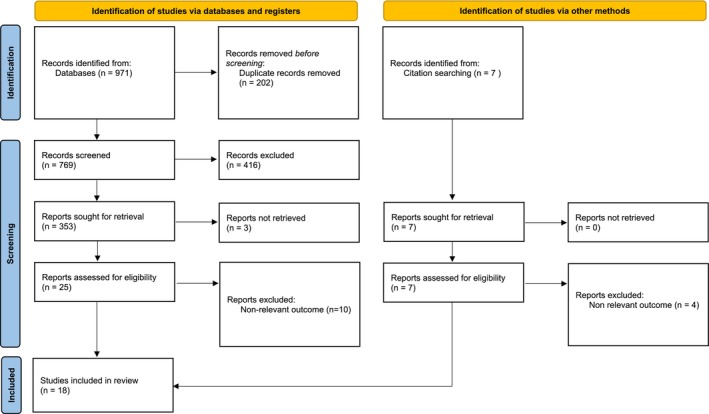
PRISMA flow diagram for study selection.

Inclusion criteria were any peer or non‐peer‐reviewed study from all health sciences literature, discussing the role of TikTok for clinical education, including the undergraduate and postgraduate setting, including all healthcare settings, conference proceedings, grey literature, reports from teaching initiatives and unpublished works available online, after TikTok's launch in September 2016. English language studies were included. Exclusion criteria were any studies not discussing the role of TikTok for clinical education or studies where the full text could not be procured (*n* = 3, Figure 3).

### 2.3 Information Sources

The Ovid platform was used to access Embase, Emcare and Medline databases. The Scopus platform, the CINAHL database via EBSCO and the Cochrane database of clinical trials were searched. References of included studies were screened to identify further relevant papers. Where full texts could not be identified online, the TikTok platform was used to reach out to content creators to access these. The search was executed on 20 January 2024.

### 2.4 Search

The following search strategy was identified: TikTok AND ((medic* OR clinic* OR health*) adj3 (educat* OR learn* OR knowledge* OR understand* OR teach*)). No formal limits were placed on the search. The search on the Scopus platform was filtered to papers including mention of ‘TikTok’.

### 2.5 Selection of Sources of Evidence

Following search, title and abstract screening was performed to identify relevant papers. Full‐text review of included papers was performed to draw out key themes from included papers relating to the review objectives.

### 2.6 Data Charting Process

A bespoke data collection tool was developed according to JBI guidance for scoping reviews [19], including study demographic data and a template for extraction of themes (Appendix S2). Data were collected by two separate researchers, with conflicts resolved by consensus.

### 2.7 Data Items

Extracted data are summarised in Table 1.

**TABLE 1:** Data items.

Data item	Details
Author	Author name
Year	Year of publication
Origin	Where the source was published or conducted
Population	Population and sample size within the source of evidence
Methods	Methodology used in presented manuscript
Intervention	Intervention type, comparator and details of these (e.g., duration of the intervention) (if applicable). Duration of the intervention (if applicable)
Outcomes	Outcomes and details (e.g., how measured) (if applicable)
Findings	Key findings that relate to the scoping review question/s

### 2.8 Critical Appraisal of Individual Sources of Evidence

Critical appraisal of included papers was performed using the relevant JBI checklist for study design [20]. Critical appraisal was performed to determine the reliability and validity of included sources of evidence. Risk of bias results were summarised and presented using the Robvis visualisation tool (Appendix S3; [21]).

### 2.9 Synthesis of Results

Key demographic data and themes relating to the review objectives were extracted. Papers were reviewed, and common and important themes were extracted and analysed to provide an overall narrative synthesis of the evidence provided.

## 3 Results

The full PRISMA checklist detailing the search strategy is included in Figure 3. Key characteristics of included papers are presented in Table 2. The results of the synthesis are summarised below.

**TABLE 2:** Characteristics of included papers.

Author	Year	Country	Aims	Population	Methods	Intervention	Outcomes
Bhuiyan, M [22]	2022	United States	Discuss the use and limitations of SoMe for professional education	Physicians	Narrative review	SoMe (Instagram, YouTube, TikTok and LinkedIn)	Use, limitations
Comp, G [14]	2021	United States	Discuss the role of TikTok in medical education	Medical educators	Narrative review	TikTok	Role, benefits, challenges
Cooper, B [23]	2022	United States	Discuss the benefits of SoMe for patient and clinician dermatology education	Dermatologists	Literature review	SoMe (Twitter, Instagram, TikTok, YouTube, and Facebook)	Use, information origins, misinformation, implications
Jarvis, N [17]	2023	United States	Discuss the use and role of SoMe for patient care, trainee education and professional development	Plastic surgeons	Scoping review	Mobile apps including TikTok	Current uses, app awareness, ethical issues
Jeyaraman, M [24]	2023	India	Discuss the role of SoMe in healthcare and medical education	Orthopaedic surgeons	Literature review	SoMe (Facebook, YouTube, Instagram, TikTok, Twitter, WhatsApp)	Role, benefits, challenges, recommendations
Kauffman, L [25]	2022	United States	Discuss the role of TikTok in radiology education	Radiologists	Content analysis	TikTok	Creation, existing content, benefits, and challenges
Lacey, H [16]	2023	United Kingdom	Make recommendations for effective production and use of TikTok in medical education	Medical educators	Literature review	TikTok	A 10‐point ‘toolbox’ for educators
Man Lin, L [26]	2022	United Kingdom	Determine the role of TikTok in FOAMed and make recommendations for research	Medical educators	Narrative review	TikTok, Instagram, Twitter	Opportunities, potential, recommendations
Morales, S [27]	2022	United States	Determine the use of virtual education to increase outreach in premedical education	Medical students	Experimental study	4–8 learning sessions using TikTok, presentations, interactive discussions	Learner assessment and capstone project
Nikookam, Y [28]	2021	United Kingdom	Discuss the role of TikTok in medical education	Physicians, Dentists	Narrative review	TikTok	Benefits, reliability, risks
Perez, M [29]	2022	United States	A physician led SoMe training programme offering formal instruction on SoMe use	Physicians, medical students	Experimental study	Presentations by peer experts	Qualitative participant feedback
Saposnik, G [30]	2023	Canada	Determine the impacts and educational value of SoMe in stroke therapies and care	Medical students	Behavioural analysis	SoMe (YouTube, TikTok, WhatsApp, Instagram)	Use patterns, engagement, attrition
Schukow, C [31]	2022	United States	Role of TikTok for pathology exposure and recruitment	Medical students	Narrative review	TikTok	Value for information sharing, education, networking
Shrivastava, S [32]	2022	India	Role of TikTok in improving medical education, public health promotion and healthcare delivery	Physicians Medical educators	Scoping review	TikTok	Role, limitations, research recommendations
Shu, H [33]	2022	Canada	Analyse SoMe medical and patient dermatology education content	Dermatologists Medical educators	Content analysis	Twitter, Instagram, TikTok	Number of medical and patient education posts
Wang, S [34]	2023	United States	Impact and challenges of SoMe for medical education, career development and research	Physicians Medical educators	Literature review	SoMe (Twitter, YouTube, Facebook, Instagram, TikTok, Reddit)	Impacts, challenges
Wang, Z [35]	2023	China	A new teaching model to bridge the gap between static knowledge and practical application	Medical students	Experimental study	TikTok for asynchronous pre‐learning and practical training course	Improvement in knowledge, skills, planning, attitudes
Jiang, X [36]	2023	United States	Discuss the potential of SoMe for pathology recruitment and education	Pathologists	Narrative review	SoMe (Twitter, Instagram, YouTube, TikTok)	Opportunities and potential benefits

### 3.1 Critical Appraisal of Included Studies

The relevant JBI checklists were used for critical appraisal of included studies [20]. Considering narrative studies, three papers were identified to have some concerns, and six were low risk. The primary area of concern is related to the lack of explanation of the relationship between text and context. The two included scoping reviews were of high concern and overall concern, respectively. Failure to appraise included studies and a lack of addressing the risk of publication bias were key areas of concern. For prevalence studies, two were of some concern, and one was of low concern. Sample size, data analysis and full description of the study subjects and setting were the most common areas of concern. Experimental studies included two with high concern and one with some concern, respectively. Failure to include control groups, appropriate statistical analysis and pre‐ and post‐intervention testing were identified as areas of concern in all experimental studies. Risk of bias was considered during analysis to inform reported findings.

### 3.2 Benefits of TikTok

TikTok has various applications in clinical education, including physician‐led training, postgraduate learning and professional development [17, 24, 29]. TikTok facilitates networking, recruitment and outreach, with benefits for both students and practising clinicians, enhancing collaboration and engagement [22, 27]. Peer interaction, tagging and content sharing promote discussion and deeper learning, encouraging academic discourse; however, concerns about misinformation remain, with reliance on clinician users to quality control available content [24, 27, 34, 35]. Its asynchronous format was reported to offer educational benefits in specific teaching contexts, including radiology and pathology [25]. Multimedia learning principles support TikTok's educational benefits, with the integration of multimedia content and interactive elements reducing cognitive load while enhancing engagement [16]. The platform's algorithm encourages repetitive content exposure, promoting knowledge retention [30, 32]. ‘Flipped learning’ using TikTok was reported to improve the preparedness of students before entering the classroom, improving synchronous learning outcomes achieved [35]. Overall, several studies reported positive findings relating to TikTok use, suggesting the platform enables rapid dissemination of medical knowledge, research and techniques, however, a predominance of low‐quality, retrospective studies reporting on this, relying on anecdotal, unvalidated evidence, limits the reliability of these findings [17, 22, 31, 36].

### 3.3 Limitations of TikTok

Evidence supporting TikTok's academic benefits is weak, with no clear impact on learning outcomes [23]. The platform lacks formal assessment tools and multimedia design standards, limiting content quality [16, 30]. Content brevity limits its use for complex clinical learning, favouring knowledge recall over deeper understanding [27, 28]. Variability in content quality persists, with misinformation risks—especially from non‐clinician creators—posing public health concerns [25, 32]. Many clinicians are unfamiliar with TikTok's educational potential, posing a barrier to engagement from clinical educators [16]. The time and resources required for content creation pose additional barriers, making institutional support crucial before wider adoption in undergraduate education may occur [16, 29]. TikTok lacks robust governance to filter inaccurate content, and the platform's algorithm prioritises engagement over accuracy, which can unwittingly expose users to unwanted, inappropriate or distressing content [29, 32]. TikTok's lack of encryption raises risks of data breaches, unintended data sharing and ethical concerns regarding informed consent and privacy [16]. The platform's algorithmic and addictive nature promotes excessive use with psychological and social consequences, and the risks of cyberbullying and exploitation with use by vulnerable users was highlighted [30, 32]. Clinician use of TikTok raised concerns over erosion of professional standards and blurring of doctor‐patient boundaries, which may influence public trust in the profession [17, 23, 24]. Clinician pursuit of influencer status and sponsorship deals may introduce conflicts of interest, prioritising virality over educational quality [26]. Current findings are largely anecdotal, requiring further high‐quality studies to validate these concerns.

### 3.4 Accessibility of TikTok

TikTok promotes FOAMed by distributing educational content, research and academic discussions globally, reducing geographic, time and resource barriers to clinical education access—especially in rural or underserved areas [17, 22, 23]. TikTok supports professional networking, education and research outreach [24, 25, 36]. TikTok analytics can help identify underrepresented groups benefiting from medical education content [32, 33, 35]. TikTok's existing popularity among learners relating to the use of short‐form videos and user‐friendly interface improves accessibility and enhance engagement with educational content [16, 18, 25]. Summarised research content may also help students engage with complex academic material, improving accessibility of academic research [36]. Experimental validation is required before the practical application of TikTok in advocacy and outreach can be accurately determined. Moreover, a prerequisite for smartphone and internet access poses ethical concerns regarding equitable access to educational content in resource‐constrained settings [29, 32].

### 3.5 Acceptability of TikTok

Clinician hesitancy towards TikTok use in education is influenced by limited SoMe fluency, lack of guidance and concerns over medicolegal implications with lack of clear ethical frameworks for use [23]. However, the COVID‐19 pandemic increased acceptance of virtual education, making platforms like TikTok more appealing for professional learning [16]. The increasing popularity of SoMe‐based FOAMed suggests TikTok could be valuable for networking, academic interaction and career development in healthcare fields [22]. TikTok is preferred over other platforms by students for outreach and communication due to its flexible and engaging format and facilitation of international and intergenerational professional and educational communication [27]. Although TikTok is increasingly used for self‐directed learning by students, use in clinical education remains underexplored, resulting in limited evidence on its effectiveness, which will limit clinician acceptance until definitive educational benefits are ascertained [17] [16].

### 3.6 Feasibility of TikTok

Several factors influence the feasibility of using TikTok for clinical educational purposes. Key concerns include the need for policies, ethical frameworks and training on data security, informed consent and e‐professionalism for physicians [22, 23]. Training in multimedia design is also essential for optimising educational value of content [16, 17, 29]. Organisational resources and funding are necessary to integrate TikTok into clinical education effectively, with peer training from existing ‘Medical Influencers’ suggested but not yet tested in real‐world settings [29, 30, 36]. Effective governance is paramount to address platform misinformation, and to ensure the quality of educational content accessed by students [22, 24]. Institutions should guide clinicians in using TikTok responsibly, ensuring professional standards and data security with the sharing of clinical data [31, 33]. Verification of accounts and transparency through conflict of interest declarations are important for content legitimacy [16]. Further qualitative and quantitative research is needed to assess TikTok's educational benefits in medical settings, including the identification of effective feedback mechanisms to support this and formally determine potential learning benefits [23, 32].

## 4 Discussion

TikTok use in clinical education has both potential benefits and challenges, but its role remains uncertain. Post‐COVID, medical education practices are evolving, and educators must be open to new technologies but approach their use with informed awareness of benefits and risks [37]. TikTok offers a large volume of accessible and engaging content, facilitating ‘micro‐learning’ and peer collaboration in undergraduate and postgraduate settings. It covers topics like basic science, clinical pathology and simulated clinical skills, allowing for tailored, self‐directed study [16]. TikTok also offers risk‐free exposure to clinical procedures, boosting confidence and competence [23]. Academic content, such as clinical study summaries, promotes greater accessibility to research, breaking down barriers like paywalls and complex language [37]. TikTok fosters professional networking, social connections and academic engagement, which can positively influence psychosocial healthcare professional well‐being [31]. Wide sharing of content enhances platform applicability to educational and academic contexts [35]. TikTok's potential for outreach and advocacy helps reduce barriers to access and encourages wider educational participation. Despite these advantages, educators remain reluctant to embrace TikTok, limiting its educational use primarily to self‐directed student learning [27]. Concerns include misinformation, lack of content quality control and insufficient use of multimedia pedagogy for optimal learning. Risks such as off‐task activity, SoMe addiction, professionalism issues and ethical concerns with ‘Medical Influencers’ using TikTok for personal gain were noted. Improved training, resources, legal guidelines and governance are paramount to address these risks.

TikTok improves learning accessibility, enabling learners to engage with material on‐the‐go, promoting self‐directed learning tailored to individual needs [14]. Mobile apps offer flexibility, personalisation and improved learning collaboration. However, the risk of distraction and off task behaviour can negatively influence learning. Educators may struggle to manage device use in classrooms, and the success of TikTok integration depends on learner maturity and motivation [15]. The need for mobile devices may also pose practical and ethical challenges in resource‐constrained environments [24]. Additionally, the changing demographics of SoMe users, influenced by geopolitical tensions and shifting platform popularity, may influence platform relevance and academic engagement [16]. Short‐form video formats suit ‘microlearning’, offering concise, engaging content; however, brevity limits depth and applicability for complex, higher‐order learning [3]. Educational use must be mindful of platform limitations to prevent misunderstanding from oversimplified content.

Misinformation risks and lack of quality control were highlighted in most included studies included. Accessing misleading or incorrect information may negatively impact learning outcomes [29]. The platform's vast, algorithm‐driven content and autoplay features increase the likelihood of inadvertently encountering poor‐quality or incorrect material [31]. This creates an ethical challenge, especially in healthcare education, where misinformation can have serious implications for patient care and professional practice, presenting a barrier to use without effective content governance [29]. Moreover, algorithmic content risk excessive use and addiction, with potential psychosocial implications, including disengagement from real‐life activities, impaired learning ability and increased rates of low self‐esteem, anxiety and depression in users [16]. The platform's short‐form content and rapid context switching can negatively affect memory, recall, execution and learning over time [27]. While TikTok may offer immediate engagement benefits, it can reduce long‐term learning effectiveness. The addictive nature of TikTok raises concerns about its ethical use in education, especially for younger students [29]. Educators must weigh the potential harms, including reduced attention spans and social well‐being, against the potential educational benefits [33]. Platforms like YouTube may be safer alternatives for educational use, especially for younger learners, while TikTok may be more viable in higher education settings, where students have more autonomy and enhanced ability to safeguarding themselves while learning in a digital space [15].

**FIGURE 4:**
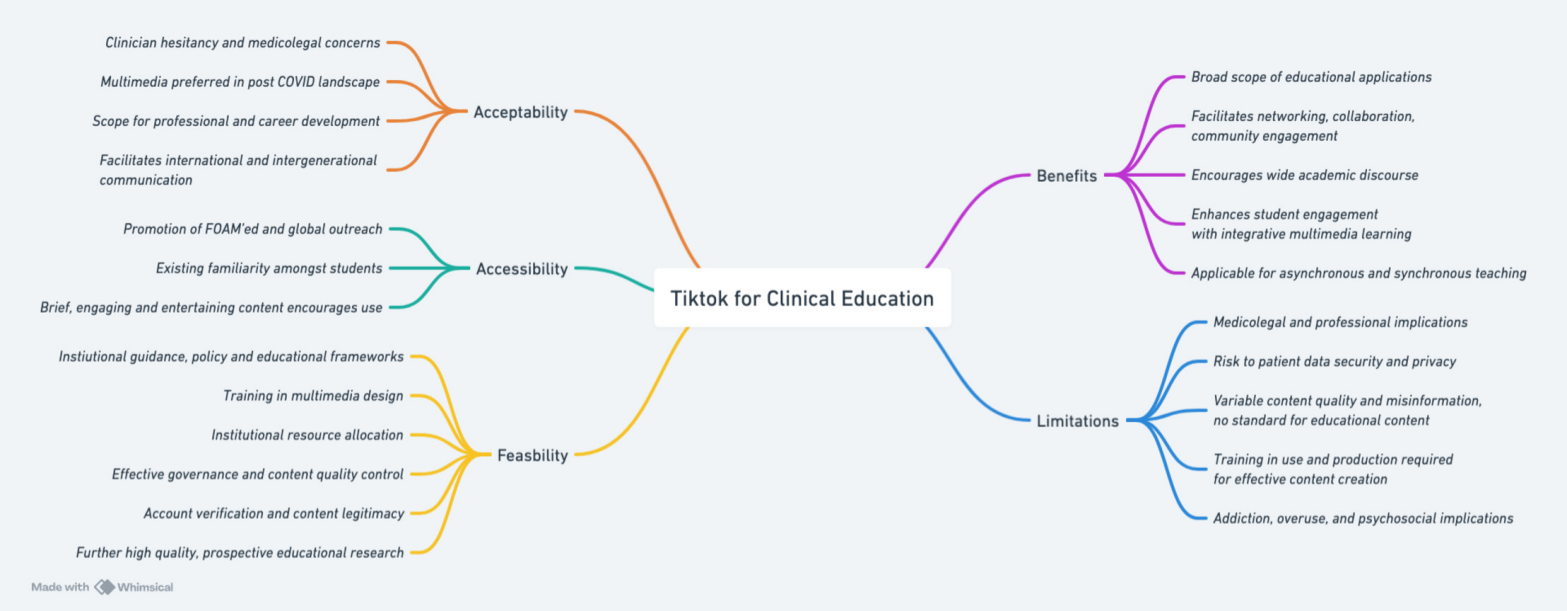
Bloom's taxonomy of learning.

There are practical challenges, such as financial and time constraints, that limit the ability of educators, especially in clinical settings, to create and identify high‐quality TikTok content [16]. The volume of content present on the platform means finding relevant, high‐quality material can be time‐consuming. Significant investment of resources, and training are additionally required to create effective TikTok content that facilitates higher learning outcomes like analysis and evaluation, which short‐form content like TikTok struggles to achieve (Figure 4) [29]. Effective use also requires awareness of multimedia design principles to avoid overwhelming cognitive load and prevent learning disengagement [3]. Many educators have competing clinical responsibilities, and the lack of funds for content development and training further hinders TikTok's adoption [29]. Without dedicated financial investment and dedicated training initiatives, integrating TikTok into clinical education may be unrealistic and potentially unethical due to the risks of ineffective learning without adequate resource development [29].

Maintaining professionalism with active clinician use of a SoMe platform and navigating appropriate interprofessional discourse on SoMe remains an individual and organisational challenge [38]. The General Medical Council (GMC) provides guidance on clinician use of SoMe, but its advice on publishing educational content is limited [39]. Clinicians must be cautious of blurring personal and professional boundaries, as misuse could have medicolegal and professional implications, considering the risks of misinformation, professionalism concerns and privacy issues [40]. Legislation relating to TikTok in different countries should also be considered; several developed nations have introduced partial bans on TikTok in professional contexts relating to privacy and security concerns, which may introduce medical indemnity and medicolegal considerations [41].

Organisations and employers are both highlighting issues and offering recommendations and guidelines relating to SoMe use in clinical settings [16, 39]. These include highlighting the importance of clinician and patient anonymity and confidentiality, appropriate patient consent for digital material sharing and the potential risks of patient and clinician online interaction [11]. Targeted clinician education on effective SoMe use remains paramount to maximise its potential for clinical education while mitigating the risks of misuse [42]. This could involve the integration of SoMe etiquette into medical school curricula to enhance clinicians with the appropriate skills to navigate the ever‐evolving digital landscape with cognizance of the potential risks [29]. Ethical and professional guidelines and legislation must be developed to avoid pitfalls including breaching confidentiality, negatively influencing patient–clinician trust and patient–clinician relationships and engaging in appropriate or unprofessional discourse and actions online [39] Institutional governance and policies on SoMe use in education should include regular feedback and support to students and educators. Developing a ‘medical e‐learning’ platform with TikTok‐style videos could mitigate some risks, such as misinformation and safeguarding concerns, by allowing content and user control [38]. However, creating such a platform faces challenges, including time and financial constraints, and requires dedicated investment and institutional support, which may limit TikTok outreach and accessibility benefits [40].

Conflicts of interest relating to TikTok for medical education include financial, professional and academic [39]. With increasing popularity of ‘Medical Influencers’, the number of creators producing and disseminating TikTok content for personal, professional and financial gain is expanding dramatically [11]. Influencers can profit from TikTok through sponsored content, advertisements and product promotions, which may lead to biased or unreliable educational content. Vulnerable users, such as students with special educational needs or poor cybersecurity, may be more susceptible to financial harm or exploitation [35]. Regulating use of professional status by clinicians on TikTok by extending GMC guidelines may help manage conflicts of interest, but this is complicated by the popularity of purported ‘Medical Influencers’ who are no longer in clinical practice [43]. Furthermore, limiting anonymity for clinicians could reduce incentives to post content, potentially affecting the quality and volume of educational TikTok material. On the other hand, maintaining clinician identification on TikTok is beneficial for career progression, networking and increasing accessibility of clinical education [39]. Balancing effective regulation with potential benefits of TikTok in expanding access to education, reducing hierarchical barriers in medicine and promoting professional development remains a major challenge [42].

Use of TikTok for medical education raises several ethical concerns, primarily related to patient confidentiality, privacy and informed consent [41]. Sharing of patient information, even with de‐identification, risks patient confidentiality and legal repercussions from data breaches. Additionally, content viewed outside its educational context may be misinterpreted, potentially leading to negative health outcomes for patients accessing content. To mitigate these risks, explicit written consent from patients before content sharing and vigilant adherence to data protection guidelines is essential when creating and sharing clinical educational material on SoMe platforms [39].

### 4.1 Limitations

There is limited observational or quantitative literature relating to TikTok for clinical education in the literature; hence, literature available to review was predominantly narrative. A lack of quantitative or qualitative data within included studies means that the evidence for TikTok benefits presented remains conceptual only, grounded in multimedia theory rather than practical educational settings. The risk of bias in available studies was significant and limited in the numerical and statistical analysis of outcomes; hence, study conclusions may be unreliable and limited in practical value. There remains a risk of publication bias in studies where authors fail to address potential challenges of TikTok use, or in experimental or prevalence studies where outcomes were presented qualitatively without quantitative analysis. There remains no definitive evidence for educators to justify TikTok use when considering the potential risks and limitations of the tool. The theoretical learning benefits of microlearning through integrative multimedia such as TikTok are understood; there remains a gap in the literature for high‐quality observational and experimental evidence determining academic outcomes in learner populations. Further experimental studies and the development of appropriate assessment tools relating to TikTok use are required before informed decision‐making about the educational use of TikTok can be made.

## 5 Conclusions

TikTok offers the potential to benefit clinical education, with potential learning benefits relating to integrative multimedia, increased content availability, accessibility of learning and widening participation and reducing barriers to access to higher education. Significant risks, including misinformation, conflicts of interest, data privacy and confidentiality, present medicolegal and legislative considerationsThe potential for SoMe to advance medical knowledge sharing and aid the development of a global knowledge exchange has been highlighted. Appropriate financial, time and resource provision for educators to optimise benefits of tools such as TikTok is required for effective uptake and to ensure the learning benefits outweigh the potential risks and consequences. This includes consideration of appropriate safeguarding measures and provision to mitigate the psychological and social implications of SoMe use in learners. Further prospective research on the benefits and opportunities of TikTok in clinical education, considering regulatory, medicolegal, financial and resource availability, is required before its definitive benefits are determined.

## Author Contributions

**Hester Lacey:** conceptualisation, writing, drafting and review. **Jim Price:** conceptualisation. **Sara Donetto:** review of manuscript and supervision. **Wajeeha Aziz:** review of manuscript and supervision.

## Funding

The authors have nothing to report.

## Ethics Statement

The authors have nothing to report.

## Consent

The authors have nothing to report.

## Conflicts of Interest

The authors declare no conflicts of interest.

## Supporting information


**Appendix S1:** Preferred Reporting Items for Systematic reviews and Meta‐Analyses extension for Scoping Reviews (PRISMA‐ScR) Checklist.
**Appendix S2:** Data collection tool.
**Appendix S3:** Critical appraisal visualisation.

## Data Availability

Data used in this manuscript are available on request.
